# Predicting the public health impact of a malaria transmission-blocking vaccine

**DOI:** 10.1038/s41467-021-21775-3

**Published:** 2021-03-08

**Authors:** Joseph D. Challenger, Daniela Olivera Mesa, Dari F. Da, R. Serge Yerbanga, Thierry Lefèvre, Anna Cohuet, Thomas S. Churcher

**Affiliations:** 1 Medical Research Council Centre for Global Infections Disease Analysis, Department of Infectious Disease Epidemiology, Imperial College London, London, United Kingdom; 2 Institut de Recherche en Sciences de la Santé, Bobo-Dioulasso, Burkina Faso; 3 Institut des Sciences et Techniques, Bobo-Dioulasso, Burkina Faso; 4 MIVEGEC, University of Montpellier, CNRS, IRD, Montpellier, France; 5 Centre de Recherche en Écologie et Évolution de la Santé (CREES), Montpellier, France

**Keywords:** Computational models, Vaccines, Malaria, Epidemiology

## Abstract

Transmission-blocking vaccines that interrupt malaria transmission from humans to mosquitoes are being tested in early clinical trials. The activity of such a vaccine is commonly evaluated using membrane-feeding assays. Understanding the field efficacy of such a vaccine requires knowledge of how heavily infected wild, naturally blood-fed mosquitoes are, as this indicates how difficult it will be to block transmission. Here we use data on naturally infected mosquitoes collected in Burkina Faso to translate the laboratory-estimated activity into an estimated activity in the field. A transmission dynamics model is then utilised to predict a transmission-blocking vaccine’s public health impact alongside existing interventions. The model suggests that school-aged children are an attractive population to target for vaccination. Benefits of vaccination are distributed across the population, averting the greatest number of cases in younger children. Utilising a transmission-blocking vaccine alongside existing interventions could have a substantial impact against malaria.

## Abstract (web summary)

Malaria transmission-blocking vaccines are in development, but roll-out strategies have not been assessed. Here, the authors show that transmission-blocking activity is likely to be higher in the field than in laboratory conditions, and that school-aged children are an important group to target.

## Introduction

Significant progress has been made in reducing the global malaria burden though in the last few years advances seem to have stalled^1^. This could be owing to a number of factors, such as funding targets not being met, mosquitoes developing resistance to widely used insecticides, or health system failures in certain regions. Malaria control is currently limited to the widespread use of insecticide-treated nets (ITNs), the spraying of insecticide indoors or in larval habitats and the use of drugs (e.g., seasonal malaria chemoprevention). Though more can be achieved by expanding the use of these existing interventions, they are unlikely to be sufficient to eliminate the disease from all areas^2^ and novel technologies are urgently needed.

At present, a variety of malaria vaccines are under development, targeting a broad range of parasite antigens^3^. Pre-erythrocytic vaccines (PEVs) target malaria parasites after they enter the human body (released from the salivary glands of a feeding *Anopheles* mosquito), before the pathogenic ‘blood-stage’ of the infection. One vaccine of this type, the RTS,S vaccine, has been evaluated in large clinical trials in sub-Saharan Africa, and has been shown to reduce the incidence of malaria in young African children, albeit with a relatively short duration of protection^4^. This vaccine is now being rolled out in a pilot implementation programme in three countries: Malawi, Ghana and Kenya where it will be given as part of an Expanded Programme on Immunisations (EPI)^5^. Vaccines which interrupt malaria parasite transmission by targeting sexual and sporogonic stages are referred to as transmission-blocking vaccines (TBVs)^3,6,7^. TBVs do not provide direct protection against infection: rather, they seek to prevent an infected human from transmitting malaria parasites to a feeding mosquito, i.e., preventing parasites from successfully infecting the mosquito.

The efficacy of any transmission-blocking intervention (TBI) is typically assessed in mosquito-feeding assays in which mosquitoes feed on infectious (gametocyte positive) blood before being dissected to check for oocysts on the mosquito midgut wall (the last parasite life-stage before the mosquito becomes infectious). The presence (prevalence) and the number of oocysts (intensity) observed with and without the intervention allow two metrics to be obtained for the intervention’s efficacy. The transmission-reducing activity (TRA) measures the reduction in oocyst counts, whereas the transmission-blocking activity (TBA) measures the reduction in the proportion of mosquitoes found to be oocyst-positive. The relationship between the two metrics is non-trivial, being strongly influenced by the ‘parasite exposure’ that mosquitoes experience, defined as the mean number of oocyst counts observed in the control mosquitoes in the assay^8,9^. For example, TBA, which is thought to be the most epidemiologically relevant metric, has been shown to decline in mosquitoes exposed to a high number of parasites^10^. This means that a TBA measured in the laboratory (where parasite exposure is typically high) may be very different to that observed in the field, whereas TRA is more consistent but not directly translatable into epidemiological impact. Another challenge in predicting TBA in the field is the variation in parasite densities observed in infections in humans: transmission from a symptomatic (or recently symptomatic) person with a high-density infection is likely to be harder to block than from one with asymptomatic low-density infection.

Any public health intervention that aims to reduce transmission must consider who contributes the most to the infectious reservoir. In the case of malaria, infectiousness needs to be determined using either membrane or direct-skin feeding assays as people with no parasites detectable by microscopy are still likely to contribute substantially to transmission^11^. Furthermore, the composition of the infectious reservoir depends not only on which age groups are most infectious to mosquitoes, but also on which age groups get bitten most frequently^12–14^.

It has been suggested that a TBV could be utilised in combination with a PEV. Adding a component to the vaccine that provides direct protection against malaria could increase vaccine up-take and, in addition to this, the combination of vaccines could be synergistic (by reducing parasite exposure, leading to a lower number of sporozoites being released from the salivary glands during the subsequent blood feed, which increases the blocking efficacy of the PEV)^15^. However, it is unclear whether combining a vaccine in a field situation would be beneficial given the complexity of malaria epidemiology.

**Fig. 1:**
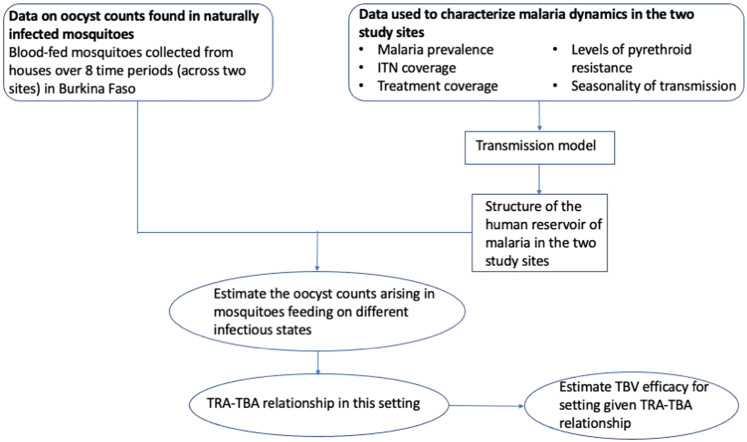
A schematic illustrating how the model relating a vaccine’s transmission-reducing activity and its transmission-blocking activity was obtained. Data on oocyst counts (Fig. 2) found in naturally infected wild mosquitoes were collected in two sites in Burkina Faso (Klesso and Longo)^21^. Retaining the disease-specific parameters fitted by Griffin et al.^27^, an established model of malaria transmission is calibrated to match the transmission intensity observed in the two trial sites. The entomological inoculation rate (EIR) is modified by changing the rate at which mosquitoes emerge so that model estimates of malaria prevalence match those observed in the trial site. It was then used to estimate the structure of the human infectious reservoir (the percentage of the population in each of the following infectious categories; treated symptomatic, untreated symptomatic, asymptomatic or asymptomatic sub-patent). This infectious reservoir could then be linked to the oocyst counts observed at the same time-point. Observed human malaria prevalence depends on the public health interventions against malaria that are in place. We used estimates of drug treatment coverage^51^ and insecticide-treated net (ITN) coverage^47^ for the region at the time the surveys were undertaken. A measure of the level of pyrethroid resistance observed in the local mosquito population was used to adjust the expected efficacy of ITNs^48^. Finally, the seasonality of malaria transmission was characterised using rainfall data^46^. The mosquito-to-human ratio was varied to best match the prevalence data for each site separately as shown in Fig. 3 and Supplementary Fig. 1. Estimates for the mean number of oocysts, resulting from mosquitoes feeding on the four different infectious states were generated by fitting to the observed population-level oocysts count data, making the assumption that mosquitoes feeding on symptomatic humans would become more heavily infected than mosquitoes feeding on asymptomatic infections. These measures of parasite exposure and how it changes over time were used to obtain the relationship between a vaccine’s transmission-reducing activity (as measured in the laboratory) and the predicted transmission-blocking activity in the field (as shown in Fig. 3E).

In this work, we introduce a modelling framework to investigate the efficacy (TBA) of a TBV against *Plasmodium falciparum* malaria in the field and estimate its public health impact when used alongside existing control interventions. Multiple TBV candidates are currently being assessed in early clinical trials^16–20^ though here we characterise a candidate TBV targeting antigen Pfs25, whose TRA as a function of antibody titre has already been estimated in a direct membrane-feeding assay using antibodies produced in mice^10^. To generate more realistic estimates of the efficacy in naturally infected mosquitoes we utilised data from wild-caught mosquitoes collected from inside houses in Burkina Faso^21^. Local entomological and epidemiological data are used to parameterise an established model of malaria transmission^22^, to estimate how TBV efficacy varies between people, how it reduces community transmission, and its overall public health impact (Fig. 1). The work examines the benefit of combining a TBV with a PEV, which age groups should be targeted for vaccination and the extent to which it can reduce residual transmission in areas with high ITN coverage.

## Results

### Estimating a vaccine’s TBA in the field

**Fig. 2:**
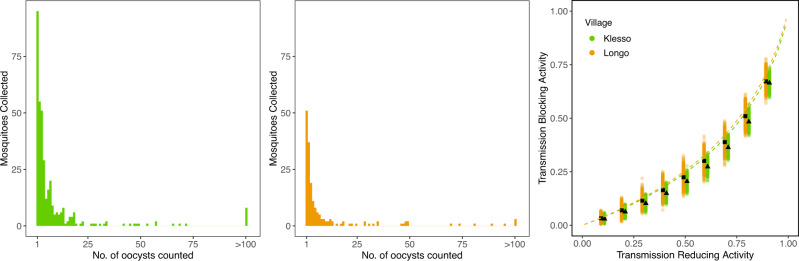
Oocyst counts measured in naturally infected mosquitoes in Burkina Faso. Wild-caught mosquitoes were collected from two villages (Klesso and Longo) and dissected to check for oocysts by microscopy^21^. In Klesso (green bars, left panel), 364 mosquitoes were positive for oocysts, compared with 179 in Longo (orange bars, middle panel). Using these oocyst counts, and a range of values for a transmission-blocking vaccine’s transmission-reducing activity, we calculated the proportion of transmission events in each site that a vaccine would block (Methods). This was done for Klesso (green) and Longo (orange), using 1000 simulations for each value of the transmission-reducing activity. In each case, the solid black marker indicates the mean value obtained for the transmission-blocking activity whilst the coloured points indicate variation from the stochastic simulations. On average, slightly higher oocyst counts were found in Klesso, which resulted in slightly lower predicted values for the TBA. Dashed lines indicate average predictions over the year for the two villages using the model outlined in Fig. 3.

To gain insight into how heavily infected naturally blood-fed mosquitoes become, we utilise data collected in two villages (Klesso and Longo) over four collection periods in highly endemic regions of Burkina Faso. In that study, mosquitoes were collected from inside houses and dissected to check for oocysts^21^. The oocyst counts observed in the two villages (Fig. 2) indicate how difficult it would be for a TBV to block these transmission events. In Klesso, oocysts formed in 18.5% (364) mosquitoes, with a mean oocyst count in those infected of 11.5 (Fig. 2A). In Longo, 7.5% (179) mosquitoes were positive for oocysts, with a mean oocyst count of 10.5 (Fig. 2B). The distribution of oocyst counts in both villages was highly overdispersed, with a number of counts >100 (8 in Klesso, 3 in Longo). Using the data from each village, we obtain a simple estimate of the vaccine’s predicted TBA from the TRA by assuming all oocysts have an equal probability of being blocked (Fig. 2C). On average, slightly higher oocyst counts were found in Klesso, which results in slightly lower predicted TBA. Though informative this simple, statistically derived estimate of TBA does not provide the full picture of human-mosquito transmission as there is a large body of evidence, which suggests people have a different probability of being infected, and those who are infected are not equally infectious^11^. To overcome this, we utilise a transmission dynamics model to facilitate interpretation of field data and predict TBV efficacy.

**Fig. 3:**
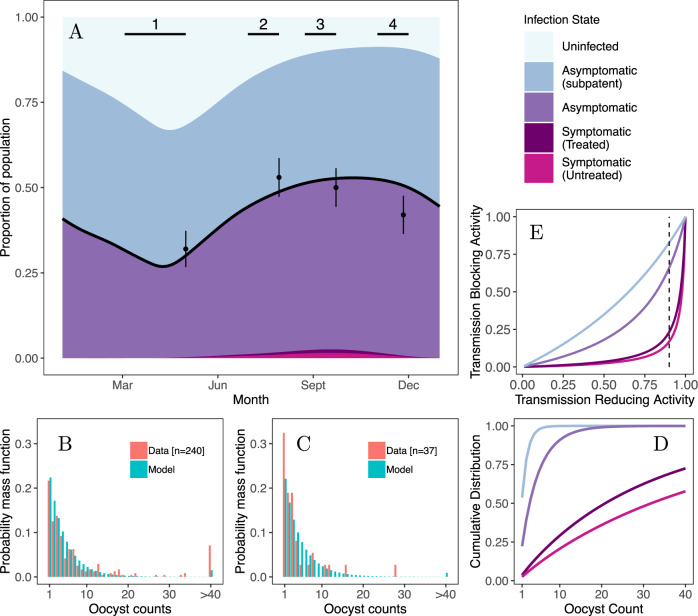
Data of malaria prevalence in humans and oocyst counts in naturally infected mosquitoes in Klesso (Burkina Faso). **A** prevalence data (black dots indicate the proportion, with the lines showing the 95% CI) collected at the end of each time period of mosquito collection. These data were used to set the transmission intensity (determined by the value of the entomological inoculation rate, here set to 490 infectious bites per-person per year) in the malaria transmission model, utilising data on ITN coverage and levels of insecticide resistance observed in the region. The black curve is the model-estimated prevalence by microscopy over time. At this level of endemicity, symptomatic infections are vastly outnumbered by asymptomatic infections, a high proportion of which are submicroscopic. The timing and duration of the four mosquito collection periods are indicated by the horizontal black lines on this panel. **B** and **C** display the distribution of oocyst counts observed in infected mosquitoes (red) in two of the four collection periods (periods 3 and 4, as indicated on the main panel), and predicted by our best-fit model (turquoise). The best-fit model was fit simultaneously to all the oocyst data, which are displayed in Supplementary Figs. 1 and 2. As the distributions are highly overdispersed, the histograms are truncated at an oocyst count of 40, and the tail of the distribution is summarised by the right-most bar. The model was fitted by attributing distinct distributions of oocyst counts for the four infectious states in the model. The cumulative distributions for the model-predicted oocyst counts are displayed in **D**: the untruncated distributions are shown in Supplementary Fig. 3. **E** illustrates the relationship (see Methods) between a vaccine’s transmission reduction activity (TRA) and transmission-blocking activity (TBA), for each of the four oocyst distributions. The dashed line in **E** indicates a TRA of 90%. The oocyst counts and malaria prevalence measured in the second study site (Longo) are displayed in Supplementary Fig. 1.

At the end of each of the four collection periods, malaria prevalence in each village was assessed by microscopy. With the aid of the prevalence data, we tailor an established malaria transmission model (as outlined in Fig. 1) to recreate the level of endemicity observed in these two sites (see Fig. 3A for Klesso and Supplementary Fig. 1A for Longo). In the model, infected humans can have four different presentations of malaria: sub-patent asymptomatic, asymptomatic, or symptomatic, with those showing symptoms either receiving drug treatment or remaining untreated. The ability of TBV candidates to block malaria transmission from a person is likely to vary substantially according to their presentation of infection.

We use the output from the transmission model to estimate the contribution of people with different presentations of malaria to the observed distribution of oocysts in mosquitoes. The proportion of people with different malaria presentations and the distribution of oocysts they cause in blood-fed mosquitoes is then used to predict the field efficacy of a vaccine (TBA). The oocyst data, along with results from the best-fit model, are shown for all collection periods in Supplementary Fig 1 (Longo) and Supplementary Fig. 2 (Klesso). Oocyst data from two collection periods in Klesso is also shown in Figs. 3B and 3C.

In Klesso, 43–51% of the population is predicted to have parasites un-detectable by microscopy over the course of the year (Fig. 3A). In Longo, where prevalence by microscopy was slightly lower (see Supplementary Fig. 1A), 31–40% of the population carry these submicroscopic infections. Our analysis (Methods) predicts that mosquitoes infected owing to feeding on these asymptomatic individuals with submicroscopic infections will become only lightly infected, having a mean of 1.6 oocysts each (1.0–5.1 CI, Fig. 3E and Supplementary Fig. 3). This distribution of oocysts means that a vaccine with a TRA of 90% is estimated to have a TBA of, on average, 83% (95% CI 67–89%, see Fig. 3E and Supplementary Fig. 3). Asymptomatic infections that are detectable by microscopy lead to slightly more heavily infected mosquitoes, having a mean of 4.4 oocysts each (4.0–5.1 95% CI, Fig. 3D and Supplementary Fig. 3), with a TRA of 90% corresponding to a blocking efficacy of 66% (95% CI 64–67%, Fig. 3E). Mosquitoes that feed on people with untreated symptomatic malaria, which in the transmission model make up a very small proportion of the population at any one time (peaking at 1.6% in the middle of the transmission season in Klesso), are predicted to become much more heavily infected, with each infected mosquito having an average of 45.9 oocysts (95% CI 33.0–54.5, see Fig. 3E and Supplementary Fig. 3). In these individuals, we predict the same vaccine to have an average TBA efficacy of 17% (95% CI 11–27%). Overall, considering how the frequency of the different infection states changes over the year and the distribution of oocysts generated by mosquitoes feeding on these individuals, we predict a TBV with a TRA of 90% to have a population average efficacy of 72%, with a maximum value of 74% during the dry season and a minimum of 70% in the middle of the transmission season (if TRA remained constant). These TBA estimates are similar to the simple estimate of TBA presented in Fig. 2C (i.e., 67% in Klesso for a TBV with 90% TRA) though the transmission dynamics model framework allows the epidemiological impact of targeting different groups with a TBV to be investigated. The model adequately captures the distribution of oocysts observed at the different time periods in the two study locations (Figs. 3B, C, and Supplementary Figs. 1 and 2), although struggles to reproduce the small fraction of oocyst counts (comprising 2% of the oocyst-positive mosquitoes) >100 oocysts per mosquito.

**Fig. 4:**
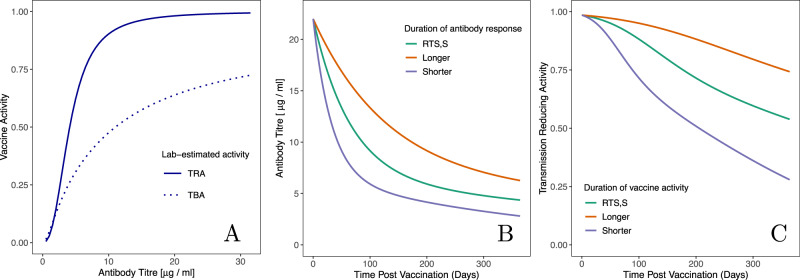
The characteristics of the hypothetical transmission-blocking vaccine candidates based on the recent activity of Pfs25 antibodies evaluated in a direct membrane-feeding assay in Burkina Faso^10^. **A** the relationship between antibody titre and vaccine activity; solid line indicates the transmission-reducing activity (TRA) whilst dashed line denotes transmission-blocking activity (TBA, average observed in all membrane-feeding experiments^10^). **B** Proposed models for the duration of the antibody response stimulated by the vaccine. The durations are motivated by antibody responses observed following vaccination with the RTS,S vaccine. For sensitivity, we also model slower and faster decays of antibody titres, obtained by halving and doubling the half-lives of both the short- and long-lasting components of the antibody response. **C** The time-evolution of the transmission-reducing activity, obtained by combing the models presented in the left and middle panels. Using the model displayed in Fig. 3E, we can translate this TRA, which depends on time since vaccination, into an expected TBA for natural transmission events.

Understanding natural exposure in this way enables us to translate the efficacy of a laboratory-evaluated vaccine into an expected efficacy in the field (Fig. 3E, see Methods for the calculation). As an example, we utilise vaccine candidate antigen Pfs25^10^ whose TRA has been described as a function of antibody titre (Fig. 4A). Fully characterising the vaccine requires estimates of the peak antibody response following vaccination, and how this response decays over time. As the duration of the vaccine’s activity is yet to be established, the well-characterised malaria vaccine RTS,S is used and compared to a shorter- and longer-lived response for sensitivity (Fig. 4B). The vaccine model displayed in Figs. 4A, B can be combined to determine how the TRA changes over time (Fig. 4C).

### Modelling the introduction of a TBV

**Fig. 5:**
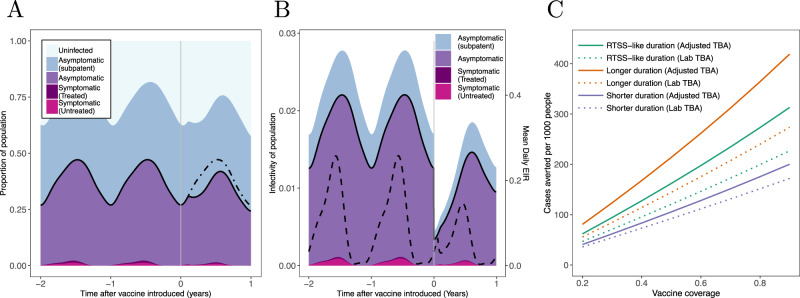
Modelling the impact of a vaccination campaign with a TBV with characteristics outlined in Fig. 4. **A** Output from the transmission model in a seasonal setting with moderate transmission (EIR of 50). The solid black curve shows the malaria prevalence of the population, measured by microscopy whilst shaded regions indicate the percentage of people with different malaria infections. Vertical grey line indicates the time of mass TBV vaccination campaign targeting all ages, with a coverage of 80%. Population prevalence is slightly reduced following vaccination (the prevalence observed in the absence of vaccination is shown by the dot-dashed black line). **B** How the infectivity of the human population changes following the same vaccination campaign. For clarity, an infectivity of (e.g.) 0.02 here means that a mosquito that feeds on a human chosen at random from the population (i.e., independently of infection status) has a 2% chance of becoming infected. The solid black curve in this panel indicates the infectivity attributable to microscopy-detectable infections. The dashed curve indicates the mean daily EIR experienced by the human population (scale given on the right-hand axis). Over the course of one year, this is nearly halved (reduced by 48%) following the vaccination. **C** The impact of the vaccination campaign on the number of clinical cases averted over the year following vaccination for campaigns with different levels of coverage and for vaccines with different durations of protection (with a shorter- and longer-acting vaccine having dot-dashed and dashed lines, respectively). All plots show vaccine efficacy as measured by TRA estimated from Fig. 4A (with TBA varying according to who is vaccinated, see Fig. 3E). For a comparison, **C** also shows results from the unadjusted TBA model (dotted lines) where TBA is consistent across all vacinees (with a TBA shown in Fig. 4A).

We illustrate the impact of this hypothetical vaccine by introducing it into a seasonal transmission setting, vaccinating 80% of the population at the start of the transmission season. Introduction of this TBV has a relatively modest impact on malaria prevalence. Prevalence by microscopy was reduced by approximately a quarter four months after the population is vaccinated, although this gain diminishes as the vaccine’s activity wanes (Fig. 5A). The impact of the vaccination campaign can be seen much more clearly when we instead show its impact on the infectious reservoir of the population (Fig. 5B). Here there is a marked decline in the infectiousness of asymptomatic individuals after vaccination. Overall, this results in a substantial reduction in overall transmission as seen by a 48% reduction in the entomological inoculation rate (EIR, the average number of infectious bites received per-person per year) over the year. Figure 5C illustrates how this reduction in transmission translates into the number of clinical cases averted due to the vaccination campaign and how this varies with vaccine coverage in this setting. A vaccine with a rate of decay equal to that of RTS,S given to 80% of the population is predicted to avert around 280 cases per 1000 people in the year following vaccination. Importantly, in this setting even low vaccine coverages are predicted to have a positive public health value with the benefit increasing linearly with vaccine coverage indicating that no minimum level of population coverage is needed for population protection. Models that take direct estimates of TBA measured in the laboratory, which do not consider the parasite exposure found in naturally infected mosquitoes, are predicted to substantially underestimate the public health impact (Fig. 5C).

To better understand the consequences of the central assumption in our TRA–TBA model—that heavily infected mosquitoes are more likely to have become infected o to feeding on symptomatic rather than asymptomatic humans—we also scrutinise a simpler model which makes the assumption that a mosquito feeding on any infectious person will, on average, develop the same number of oocysts. A single probability distribution is fit to the observed oocyst counts that is then used to estimate the relationship between TRA and TBA for all individuals. We show the results obtained in Supplementary Figs. 5A and 5B, alongside results from the more complex model. The alternative TRA–TBA relationship is very similar to that obtained for asymptomatic patent infections in the more complex model. This is not surprising given these lightly infectious individuals make the largest contribution to the infectious reservoir. The greater public health impact of a TBV is predicted by the more complicated model (Supplementary Fig. 5C) as in this model the TBV is highly effective at blocking transmission from people carrying long-lasting, low-density infections. For the remainder of this paper, we will use the more complicated model; however, we stress that the assumption behind this model should be tested with more data from a range of transmission settings.

### Identifying key age groups to vaccinate

**Fig. 6:**
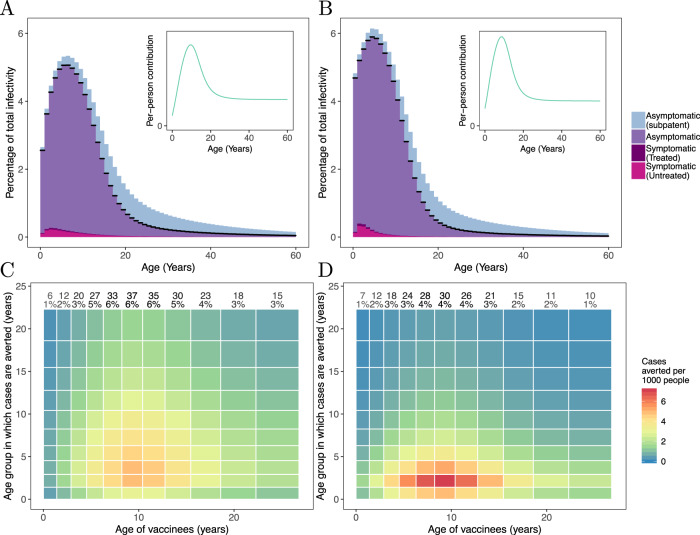
Determining which age groups to target for vaccination with a TBV. Upper panels: stratifying the infectivity of a malaria-endemic population by age. In a seasonal setting, without any active interventions, we calculate the average infectivity of the population over one malaria transmission season for a low transmission setting (**A**, EIR=15), and a high transmission setting (**B**, EIR=100). In the high transmission setting, the mean infectivity of the population over this time period was 0.029 compared with 0.018 in the low transmission setting. In these panels, we bin the population into 1-year age groups and show the contribution that each age group makes to the total infectivity. The black line on each bin indicates the infectivity attributable to microscopy-detectable infections. At the higher transmission intensity (**B**), infectivity is more concentrated in younger age groups. The inset in each figure shows the per-person contribution to the force of infection from humans-to-mosquitoes, accounting for the population demography (an important component in the population’s infectivity) and age-dependent biting rates. Lower panels: Identifying key age groups to target with a transmission-blocking vaccine. Here, we ran multiple simulations, in each instance only vaccinating an age group that comprised one-fifteenth of the total population (*x* axis). Colour denotes the number of clinical cases averted in the year following vaccination for each age groups (*y* axis). The total cases averted per 1000 people in this time period is displayed in text at the top of each column, along with the percentage of clinical cases averted by the vaccination campaign. We show results obtained for the same transmission settings used in the upper panels (EIR=15 in **C**, EIR=100 in **D**). When transmission is high, cases averted are concentrated in younger age groups. In each setting, school-age children (5–15 years) constitute a key population to target with a transmission-blocking intervention.

It is important to consider which age groups contribute most to transmission and, therefore, should be prioritised in a TBV vaccination campaign. In malaria-endemic settings, of both high and low transmission intensity, the modelling indicates that the most infectious individuals are concentrated in younger age groups (Figs. 6A and 6B). However, the contribution that adults make to onward transmission is amplified as they are bitten more frequently than young children^14^. The per-person contribution to onward transmission (insets of Figs. 6A and B) is linked to the population’s infectivity via the demography of the setting, and the age-dependent biting rates. In these two settings, results generated by the transmission model indicate that school-aged children infect the most mosquitoes. Younger children also contribute to transmission, in part because they are more numerous in the community. We use the transmission model to examine the impact of targeting different age groups for vaccination, as well as monitoring the age groups in which the burden of malaria is reduced. In the two settings considered here, we find that vaccinating children of ~10 years of age averts the highest number of cases. For example, in a high transmission setting, our modelling work suggests that vaccinating children of 8–10 years of age could avert almost twice as many cases as vaccinating the same number of 2–4-year-olds or 15–18-year-olds (Fig. 6D). The benefit of a TBV can be spread quite diffusely through the population though the greatest reduction in the number of cases is found in pre-school children, especially in higher transmission settings.

### Utilising a TBV in addition to ITNs

**Fig. 7:**
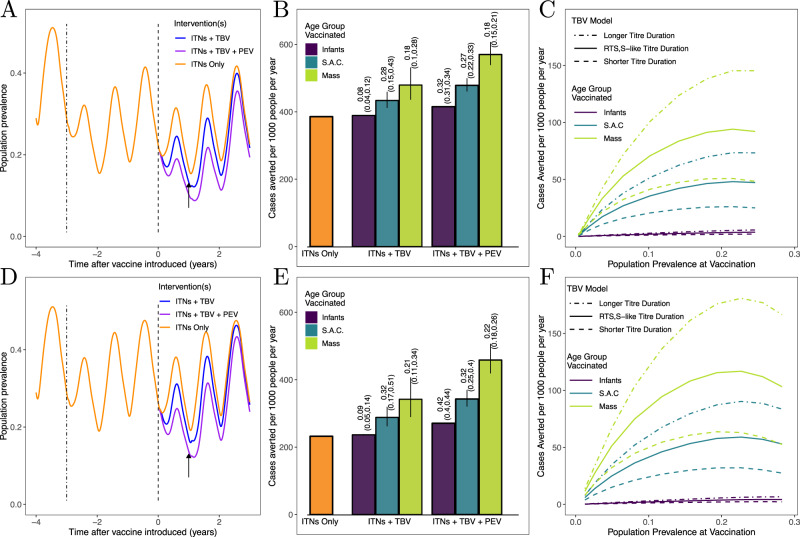
Utilising a malaria vaccine alongside insecticide-treated nets (ITNs), in a setting where ITNs are fully effective (top panels) and in a setting with high levels of insecticide resistance in the mosquito population (lower panels). **A** and **D** An illustration of the scenario being modelled. ITNs are introduced (dot-dashed line) into an area of high, seasonal malaria transmission (EIR=100), at a coverage of 60%, and are replaced every 3 years. The second distribution of ITNs coincides with a pulsed vaccination campaign. A TBV is administered, with and without a PEV. In all vaccination campaigns, a booster dose is delivered after 12 months (timing indicated by the black arrow). **B** and **E** cases averted by a vaccination campaign. Here, we show cases averted per year due to the distribution of ITNs (orange bar), and how additional cases can be averted by a vaccination campaign (introduction shown by the dashed line  in the left panel). We explored three target populations: infants (0.5–2.5 years of age), school-aged children (5–15 years old) and a mass vaccination campaign (everyone over 6 months of age). In each scenario, a vaccine coverage of 80% of the target population was used. We show how the impact of a TBV (administered with and without a PEV) varies with the duration of the vaccine’s activity (displayed in Fig. 4), indicated by the vertical black lines on each bar. The cases averted are adjusted for the number of years each intervention is active: six for the ITNs, three for the vaccine(s). The numbers above each bar show the cases averted per vaccine regimen administered. **C** and **F** How vaccine impact varies with the level of malaria endemicity, which we represent by the population prevalence at the time the TBV is introduced. Here, we show cases averted in addition to those averted by ITNs, varying the annual EIR (experienced prior to the introduction of bednets) from 1 to 150. These panels underline the potential for a longer-lasting TBV to avert a greater number of clinical cases. The impact of the combined vaccination campaign as transmission intensity is varied is shown in Supplementary Fig. 4.

Any novel intervention against malaria is likely to be introduced into areas where other malaria control interventions are already present. In particular, ITNs have been widely distributed in sub-Saharan Africa and are likely to remain the primary element of malaria control in the near future. Even if a high proportion of a population is sleeping under an effective ITNs most regions are likely to have ongoing transmission, which a TBV can help reduce. This scenario is illustrated in Fig. 7A, where bednets have been introduced into a high transmission, seasonal setting followed 3 years later by the introduction of a TBV (an initial round of vaccination, followed by a booster dose 12 months later). Mass distribution of a TBV (with an duration equal to that of RTS,S) is predicted to avert an additional 87 malaria cases per 1000 people per year over the three year period compared to bednets alone (at 0.18 cases per vaccine regimen delivered, Fig. 7B). We also assess the impact of administering a TBV to very young children (0.5–2.5 years) using the model. This age range was chosen to broadly capture the impact of introducing a vaccine into an EPI programme, in the way that RTS,S is being delivered in the current pilot implementation programme^5^. In the model, utilising a TBV in this way has minimal impact, in part because the contribution to the infectious reservoir from this age group is low as very young children are not bitten as frequently as older children or adults. Targeting school-age children achieves good results, averting 48 cases per 1000 people per year and doing so more efficiently (with 0.28 cases per vaccine regimen delivered, Fig. 6B). It has been suggested that it may be practical to combine a TBV with a PEV to enhance control. Doing so substantially increases the number of cases averted across all treatment strategies, with a vaccination campaign containing both vaccines and targeted at school-aged children averting 94 cases per-person per year (in addition to cases averted by ITNs).

We show that the absolute number of clinical cases averted will depend on the endemicity of transmission, with the vaccines typically having a higher public health impact in areas of higher transmission, although impact does saturate at very high transmission intensity (Fig. 7C). Furthermore, the impact of vaccination is likely to be greater if ITN effectiveness is reduced owing to the spread of mosquitoes resistant to the insecticides on nets, which is increasingly widespread across Africa (Fig. 7D–F)^23^. In such a scenario, we find that the number of cases averted by administering a TBV to school-aged children increases to 56 per 1000 people per year (see Fig. 7E). This represents an increase of 17%, compared with a setting with no insecticide resistance.

## Discussion

TBVs are predicted to have a substantial public health impact though this will vary depending on where they are introduced and who is vaccinated. Thus far, these vaccines are mainly evaluated using membrane-feeding assays in the laboratory, it is unclear how effective they would be in field settings. Here we attempt to predict field efficacy before a candidate passes down the development pathway. The prospective nature of the work means that many simplifying assumptions about the candidate vaccine, its deployment and the process governing transmission need to be made. Many of these assumptions will need to be refined in the future as understanding increases through the exercise provides a flexible framework on which future candidates can be evaluated to facilitate the discussion on how such vaccines could, in practise, be utilised.

A number of candidate TBVs are currently in development, some of which have been tested in malaria-endemic settings (see e.g., ref. ^20^.). It is hoped that, in the next few years, data from TBVs trialled in malaria-endemic settings will be available to parameterise models of the activity & longevity of candidate vaccines. Such data may also aid the assessment of which parasite antigen (or combination of antigens) is the most attractive for targeting with a vaccine. In the absence of data for the duration of vaccine activity, we make the conservative assumption that antibodies will persist in a similar manner to RTS,S. It is hoped that future TBVs will have a longer duration of activity, so predictions of public health impact are like to be conservative. Nevertheless, many of the qualitative conclusions drawn will be consistent irrespective of the characteristics of the vaccine in question.

The modelling framework developed here allows the incorporation of the relationship between TRA (which is typically estimated in the laboratory) to TBA (which is believed to have the most epidemiological significance in the field) into a transmission dynamics model. Utilising data from Burkina Faso we show how a vaccine candidate, which only had a TBA of 50% in the laboratory is likely to have a TBA of ~72% in field settings where the majority of infected mosquitoes are likely to develop low numbers of oocysts. Membrane-feeding assays typically expose candidate interventions to very high levels of parasite exposure, in order to obtain large numbers of infected mosquitoes. The consequence of this is that mosquitoes often develop higher numbers of oocysts than found among wild-caught mosquitoes (see e.g., ref. ^24^). For example, in the DMFAs which informed the TRA model used here, the parasite exposure was 32 (averaged over a number of experiments)^10^. This work shows that models that fail to account for this relationship are likely to substantially underestimate vaccine impact. Here, we parameterise the model for a setting with a mean burden of 2.2–14.7 oocysts per mosquito (the mean oocyst counts among infected mosquitoes collected in each study period in each site). This is likely to be the one of the higher estimates observed in the field, so these experiments need to be extended to other areas with lower disease endemicity where TBA is likely to be even higher.

Incorporating the hypothetical TBV into a detailed model of malaria transmission enables us to assess the key age groups to target and demonstrated the potential impact of utilising a TBV alongside existing interventions against malaria. It has been suggested that a TBV could be included within an extended infant immunisation programme. This work suggests that unless the vaccine has a long duration of activity this strategy is unlikely to be successful. This is in part because malaria prevalence peaks in slightly older children (e.g. refs. ^25,26^) even in areas of high transmission, and also because very young children are not bitten with the same frequency as older children or adults. Instead, the model suggests a TBV should be targeted toward school-age children who are more likely to carry long-lasting asymptomatic infections against, which the TBV would be more effective. This is consistent with community-level assessments of the human infectious reservoir^13,14^. Moreover, the modelling underlines the fact that any community-based intervention focusing on transmission will convey the greatest benefit to young children who have the highest burden. As Fig. 5 shows, this is true regardless of the age group vaccinated. Model predictions should be treated with caution as there is still considerable uncertainty in who contributes the most to mosquito infection. The transmission model^22,27^ was parameterised to age-stratified prevalence and incidence data from across sub-Saharan Africa, as well as community-level infectivity studies^28–30^, but how the infectious reservoir changes between settings with different transmission intensities with different levels of vector control is still poorly understood. There is clear evidence from field studies that school-aged children are a key population to target to reduce transmission at the community level^13,14,31^ but what exact age range to target requires further scrutiny once the characteristics of a future TBV becomes better understood.

The transmission model utilised here has no spatial structure, which means that a mosquito that becomes infected will not preferentially return to the same individual or household for its next feed. In the model, therefore, vaccinated individuals do not receive any additional benefit from the vaccine, compared with those who are not vaccinated. Further work is needed to understand the fine-scale spatial structure of who-infects-whom as there is likely to be some geographical structure as mosquito flight is likely to be predominantly local and choice between human hosts presumably heterogeneous. This will have implications for clinical trials attempting to measure a reduction in prevalence or incidence following administration of a TBV. The model also assumes that mosquitoes are equally infectious irrespective of the number of oocysts that formed. Laboratory data have suggested that lightly infected mosquitoes are less infectious^32^ though other controlled human malaria infections have failed to find this association^33^. If this were the case, a partially effective TBV that reduced oocyst density but failed to clear the parasite may have a greater impact than currently predicted.

The modelling framework should be further refined as we understand more about the processes governing transmission and the characteristics of lead TBV candidates. Here, we have used a compartmental model, which captures vaccination campaigns as a pulsed intervention, treating everyone at the same time. For vaccines used as part of an EPI programme, it is more realistic to vaccinate infants upon reaching a certain age. More detailed, individual-based modelling can be used to reflect this and capture changing estimates of coverage of different interventions, as well as issues such as reduced compliance between initial and booster doses^34^. In future, that type of model parameterised with clinical trial data could be used to estimate the feasibility of using a TBV to interrupt malaria transmission in different settings.

As was observed with the RTS,S cluster-randomised control trial^4^, the addition of a TBV to a community who is already partially protected by ITNs diminishes the number of cases averted owing to the vaccine. In reality, ITN use is likely to be lower in non-trial settings and so the public health benefit of an intervention like a vaccine, which does not require continued user-compliance is likely to be larger. Here, we assumed that people continue to use nets for three years when usage is likely to decline over this time period. The majority of simulations carried out also assumed ITNs are working effectively. This is unlikely to be the case as mosquitoes resistant to the pyrethroid insecticide used on ITNs are now widespread across Africa and all ITNs currently recommended by the World Health Organisation contain pyrethroids as their active ingredient. Simulations show that the impact of the TBV is higher in areas of high pyrethroid resistance and the vaccine can help mitigate the loss of control, especially as it can prevent transmission outside the home where people are often exposed^35^.

Unless a TBV with a much longer duration of activity than the one investigated here can be developed, it is unlikely that TBVs alone will be a ‘silver bullet’ for malaria control. Nevertheless, we have demonstrated how a TBV could be a valuable tool when used in consort with other public health interventions in the battle against malaria. There is increased interest in other transmission-reducing strategies such as the use of drugs to reduce infectivity^36^, endectocides to kill blood-feeding mosquitoes^37^, or by modifying the mosquito vector through symbionts or genetic modification^38^. Many of the issues associated with TBVs will be similarly applicable to these other transmission targeting interventions. Frameworks like the one developed here can be used to estimate the public health impact of these new interventions and can be used to justify future development and support their evaluation in the field.

## Methods

### Summarising the transmission model

A number of different dynamic transmission models of malaria have been developed and calibrated to data from malaria-endemic settings^27,39–42^. These models differ structurally (e.g., how immunity is acquired), and they are informed by different data sources. As a result, predictions do vary, as demonstrated in two studies in which multiple models were used to predict the impact of the RTS,S vaccine and mass drug administration^43,44^. Nevertheless, the models are qualitatively similar, in the sense that they predict that clinical incidence becomes more concentrated in younger age group as transmission intensity increases, and that malaria prevalence saturates at very high transmission intensities^43^. The model used here is a deterministic, compartmental model developed by Griffin et al.^27^, which has been fitted to age-stratified data on prevalence and clinical incidence of malaria collected in a range of transmission settings across sub-Saharan Africa. It allows for heterogeneity of transmission, age-dependent biting rates, and the development of naturally acquired immunity in populations exposed to malaria. The transmission model is initially used to estimate the relationship between malaria prevalence and the reservoir of infection in mosquitoes by parameterising it using data collated on the two study sites. It is then modified to include a TBV to predict its public health impact in different scenarios.

The transmission model is summarised in detail in the Supplementary Methods and briefly described here. A schematic of the model structure is provided in Supplementary Fig. 6. When malaria-free individuals (denoted *S* for susceptible) become infected, they can develop clinical disease or become asymptomatic carriers of the parasite (state *A*), depending on their prior exposure to malaria. The clinical disease can either be treated (state *T*) or remain untreated (state *D*). If treated, individuals recover, pass through a period of drug prophylaxis (state *P*) and return to being susceptible. If untreated, individuals remain symptomatic for a period of time, then harbour a long-lasting asymptomatic infection. Initially, this infection will be detectable by microscopy (state *A*) before becoming submicroscopic (state *U*). Individuals who do not develop symptoms also progress from state *A* to *U*. In higher transmission settings, for some individuals with a very high level of prior exposure, the infection may be submicroscopic for its entire duration. After the infection is cleared, individuals return to state *S*, although it is possible to acquire an additional infection whilst carrying an asymptomatic infection (which can lead to the individual becoming symptomatic again, depending on prior exposure). In four of the states (*D*, *T*, *A* and *U*), individuals are capable of infecting a feeding mosquito. The mosquito population is also included in the model, with adult mosquitoes stratified by malaria status (susceptible, exposed, infectious, see ref. ^45^. for full details of the mosquito component of the model).

We denote the probability of a human in state *X* infecting a feeding mosquito as *c*_*X*_. In the transmission model^22^, *c*_*D*_ > *c*_*T*_ > *c*_*U*_. Parameter *c*_*A*_ has a peak value equal to *c*_*D*_, but its value diminishes towards that of *c*_*U*_ in sub-populations with high immunity (where the parasite densities of infections are lower and, therefore, transmission of parasites to a mosquito is less likely). In this work, we will represent the effect of a TBV through a reduction of values of these parameters in the vaccinated population as follows,

1 $$c_X^\prime = c_X\left( {1 - TBA_X\left( z \right)} \right),X = \left( {U,A,T,D} \right),0 \le z \le 1,$$

where *c’*_*X*_ is the human-mosquito transmission probability of a vaccinated individual in state *X* and, *TBA*_*X*_*(z)* is the TBA from infections in state *X*, given a TRA of *z*.

### Oocyst counts found in naturally infected mosquitoes in Burkina Faso

We use data on oocyst counts observed in wild-caught *Anopheles* to assess how heavily infected mosquitoes become after feeding naturally in the wild. Let *m*_*X*_ denote the average number of oocysts that successfully develop in a mosquito following a blood meal from a human host in infection state *X*. We utilised data collected in two villages (Klesso and Longo) close to Bobo-Dioulasso in Burkina Faso, over four time periods in 2014 (making eight data points in total)^21^. Using a mouth aspirator, fed mosquitoes were collected early in the morning from inside houses. All collected mosquitoes were transported to IRSS laboratory where *Anopheles* females were selected and maintained in an insectary for 7 days before dissection under a microscope to count the number of oocysts. This time period was selected so that all oocysts from previous blood-meals are likely to have burst whilst infections from the last blood meal will remain visible (though see ref. ^21^. for an alternative method of estimating parasite exposure). Hence, broken oocysts were not included in the analysis here, although they were counted in the original study.

At the end of each period of data collection in each site, malaria prevalence in the village was estimated using a sample of 300 people (100 people under 10 years old, 100 people between 10 and 20 years old, and 100 people over 20 years of age). Results are shown in Fig. 3 for Klesso and Supplementary Fig. 1 for Longo. The transmission model is used to estimate the proportion of the human population in the different states of malaria infection (*D*,*T*,*A* and *U*) in the field site at the time of data collection. This is undertaken by characterising the region according to the seasonality of mosquito abundance^45^ (as estimated from rainfall patterns^46^), ITN usage (as estimated by the Malaria Atlas Project^47^), and levels of insecticide resistance in the local mosquito population (which influences ITN efficacy, as estimated from a discriminating dose bioassay^48^). We then calibrate the model to match the observed malaria prevalence data by adjusting the mosquito-to-human ratio in each site. Least squares methodology is used to fit model predictions of malaria prevalence at the different time points (as detected by microscopy, states *D*, *T* and *A*) to the observed cross-sectional data from the appropriate survey (Fig. 3 and Supplementary Fig. 1). All other disease-specific parameters in the transmission model were as previously estimated (Supplementary Table 3) (27).

To model the oocyst counts observed in Burkina Faso, we associate a probability mass function for the oocyst count obtained from a successful feed on an individual in each of the infectious states in the model (*U*, *A*, *T*, *D*). We assume that each oocyst count is the result of a single blood meal, and that the high oocyst counts stem predominantly from mosquitoes feeding on individuals who are, or have recently been, symptomatic. The latter assumption implies that we do not expect to observe many highly infected mosquitoes when there are few symptomatic infections in the human population (i.e., during the dry season). This argument is rather heuristic but is consistent with the observed data and allows us to account for the overdispersed nature of the oocyst counts. Furthermore, it enables us to assess the consequence that this overdispersion will have for a TBV, i.e., some transmission events are easier to block than others.

In the transmission model, the force of infection from humans-to-mosquitoes contains a contribution from the proportion of the human population found in each of the four states. The force of infection reflects that fact that the four malaria states in the model are not equally likely to infect a mosquito, and it is adjusted for heterogeneity of transmission and age-dependent biting rates. The average number of oocysts developing in a mosquito having fed on someone of state *X* is denoted by *m*_*X*_. We will employ the negative binomial distribution which, for mean *m* and dispersion parameter *k*, has the general form

2 $${\uppi}\left( {i;m,k} \right) = \frac{{\left( {k + i - 1} \right)!}}{{i!\left( {k - 1} \right)!}}\left( {1 + \frac{m}{k}} \right)^{ - k - i}\left( {\frac{m}{k}} \right)^i,i = 0,1,2,3, \ldots .$$

As we seek to characterise the oocyst counts in successfully infected mosquitoes only, we use a truncated negative binomial (denoted $\widehat \pi$). We write the probability of a mosquito developing *i* oocysts after feeding on a human in state *X*

3 $$\widehat {\uppi}(i;m_X,k_X) = \frac{1}{{1 - {\uppi}\left( {0;m_X,k_X} \right)}}{\uppi}\left( {i;m_X,k_X} \right),i = 1,2,3, \ldots$$

Our composite distribution (denoted by the subscript *) that we fit to the data is $\widehat {\uppi}_ \ast ^{ab}\left( {i;m,k} \right)$

which includes weighted contributions from the four infectious states. The weighting will vary depending on the time period *a* = (1, 2, 3, 4) and study site *b* = (1,2). For simplicity, we use the same dispersion parameter for each infectious state, hence:

4 $$\widehat {\uppi}_ \ast ^{ab}\left( {i;m,k} \right) = w_D^{ab}\widehat {\uppi}_D\left( {i;m_D,k} \right) + w_T^{ab}\widehat {\uppi}_T\left( {i;m_T,k} \right) + w_A^{ab}\widehat {\uppi}_A\left( {i;m_A,k} \right) + w_U^{ab}\widehat {\uppi}_U\left( {i;m_U,k} \right).$$

Here we are only concerned with the proportion of the human population that is infectious, hence we normalise to ensure that *w*^*ab*^_*D*_*+ w*^*ab*^_*T*_ *+ w*^*ab*^_*A*_ *+ w*^*ab*^_*U*_ =1, for each combination of *a* and *b*. We denote the data points as ***d***^***ab***^ and define *S*^*ab*^ as the number of data points at each time point and location. We can then write the likelihood of the data for a given set of parameters ${\uptheta} = \left( {m,k} \right)$ as

5 $$L\left( {D|{\uptheta}} \right) = \mathop {\prod }\limits_{b = 1}^2 \mathop {\prod }\limits_{a = 1}^4 \left[ {\mathop {\prod }\limits_{j = 1}^{S^{ab}} \widehat {\uppi}_ \ast ^{ab}\left( {d_j^{ab};{\uptheta}} \right)} \right].$$

We introduce prior distributions to ensure that higher oocyst loads are attributed to symptomatic, rather than asymptomatic individuals. Single estimates of *m*_*D*_, *m*_*T*_, *m*_*A*_, *m*_*U*_ and *k* are fit for the entire dataset using the Metropolis–Hastings algorithm. Supplementary Table 1 summarises the posterior distribution obtained.

### Developing a relationship between TRA and TBA in naturally infected mosquitoes

Estimates of the single-feed infection load experienced by mosquitos feeding on a person of malaria state *X*, are used to understand the relationship between a vaccine’s TRA and TBA in natural infections. In a naturally infected mosquito that, without the presence of the vaccine-induced antibodies, would develop *i* oocysts, we assume that we can use the TRA (which here we denote as *z*, where 0 ≤ *z* *≤* 1) to calculate an independent probability of blocking the formation of each oocyst. Using the binomial distribution, the probability that zero oocysts are formed is *z*^*i*^ (as shown in Fig. 2; this relationship is independent of the TBV model used). Considering the four fitted probability mass functions in turn (see Supplementary Fig. 3), we can calculate the desired TBA by summing over the distribution i.e.,

6 $$TBA_X\left( z \right) = \mathop {\sum }\limits_{i = 1}^\infty z^i\widehat \pi \left( {i;m_X,k} \right),X = \left( {U,A,T,D} \right).$$

We can proceed by noting that this infinite sum is closely related to a probability generating function^49^. For the negative binomial distribution in the form used here, the probability generating function *G(z)* is

7 $$G\left( z \right) = \mathop {\sum }\limits_{i = 0}^\infty {\uppi}\left( {i;m,k} \right)z^i = \left( {\frac{k}{{k - m\left( {1 - z} \right)}}} \right)^k.$$

As we are using a truncated distribution, some algebraic manipulation is required to obtain the desired TBA:

8 $$\begin{array}{l}TBA_X\left( z \right) = \mathop {\sum }\limits_{i = 1}^\infty \widehat {\uppi}\left( {i;m_X,k} \right)z^i = \frac{1}{{1 - \left( {\frac{k}{{k + m_X}}} \right)^k}}\left[ {\left( {\frac{k}{{k + m_X\left( {1 - z} \right)}}} \right)^k - \left( {\frac{k}{{k + m_X}}} \right)^k} \right],\\ X = \left( {U,A,T,D} \right).\end{array}$$

The removal of the infinite sum means that, for a given negative binomial distribution, we can generate an analytical expression for the relationship between the TRA, which will vary with the time elapsed since vaccination, and the estimated TBA for people with different malaria states in the model (Fig. 3E). In this way, the overall TBA for a given setting depends on the relative frequencies of the four infectious states in the model. The schematic shown in Fig. 1 illustrates how the different sources of data were used alongside the transmission model to obtain a relationship between TRA and TBA in the field.

### Vaccine model

No TBV candidate has yet progressed through Phase III control trials though there are a number of lead candidate antigens^3^. As an illustrative example of a TBV, we utilise a relationship obtained between antibody titre and TRA using antibodies against Pfs25^10^. Antibodies were produced in mice, and their transmission-blocking efficacy was obtained via direct membrane-feeding assay (following serum-replacement), with parasites obtained from infected children in Burkina Faso, using *Anopheles coluzzii* mosquitoes. The mosquitoes were dissected 7 days after blood-feeding to examine for the presence of oocysts on their midgut. An estimate of the TRA from each parasite source was obtained by comparing oocyst counts obtained with and without the presence of the TBV-induced antibodies.

The rate of antibody decay will influence the duration of activity of any TBV. In the absence of this information, we selected the same rate of loss of antibodies as was estimated for the RTS,S vaccine. The antibody dynamics of that vaccine have been modelled in terms of a short-lived and long-lived response^50^ (see Supplementary Methods and Supplementary Table 2). Given the uncertainty in the duration of a TBV, we also conducted a sensitivity analysis on this value by either halving or doubling both the short- and long-term responses, as displayed in Fig. 4. Modelling scenarios that also included a PEV in the vaccination campaign utilised a published model of RTS,S (described in Supplementary Methods)^34,50^. We assume that the antibody titres generated following vaccination of older children or adults are similar to those observed in the Phase III trial in infants and young children. Previous animal models have indicated that there might be a synergistic interaction between these vaccines, regulated by parasite density^15^. Here, we assume that the vaccines are independent of one another. The transmission model contains no spatial structure, so the benefit of a TBV is dissipated across the whole population. Similarly, the use of ITNs and vaccines is considered to be independent with coverage consistent across all age groups (unless a certain age group is specifically targeted for vaccination). The transmission model utilises an exponential distribution (i.e., risk of mortality is constant) for the age structure of the population, parameterised using data from Tanzania, with an average life-expectancy of 21 years^22^.

### Reporting summary

Further information on research design is available in the Nature Research Reporting Summary linked to this article.

## Supplementary information

Supplementary Information

Reporting Summary

## Data Availability

The oocyst data are available from the Dryad repository (10.5061/dryad.9p8cz8wfh).

## Code availability

The R scripts used to generate the results reported in this paper are available on Github: https://github.com/JDChallenger/Malaria_TBV.
