# Supplementary material for: Predicting the public health impact of a malaria transmission-blocking vaccine
Source: Nat Commun. 2021 Mar 8;12:1494. doi: 10.1038/s41467-021-21775-3 (PMC7940395; doi:10.1038/s41467-021-21775-3)
Supplement: Supplementary file 1 — Supplementary Information [file 41467_2021_21775_MOESM1_ESM.pdf]

Predicting the public health impact of a malaria transmission-blocking vaccine

Joseph D. Challenger[1], Daniela Olivera Mesa[1], Dari F. Da[2], R. Serge Yerbanga[2,3], Thierry Lefèvre[4,5], Anna Cohuet[4], Thomas S. Churcher[1]

1. Medical Research Council Centre for Global Infections Disease Analysis, Department of Infectious Disease Epidemiology, Imperial College London, London, United Kingdom

2. Institut de Recherche en Sciences de la Santé, Bobo-Dioulasso, Burkina Faso

3. Institut des Sciences et Techniques, Bobo-Dioulasso, Burkina Faso

4. MIVEGEC, University of Montpellier, CNRS, IRD, Montpellier, France

5. Centre de Recherche en Écologie et Évolution de la Santé (CREES), Montpellier, France

SUPPLEMENTARY INFORMATION

This document contains:

1. **Supplementary Figures 1-6**
2. **Supplementary Methods**
3. **Supplementary Tables 1-3**

All references are numbered as per the main text.

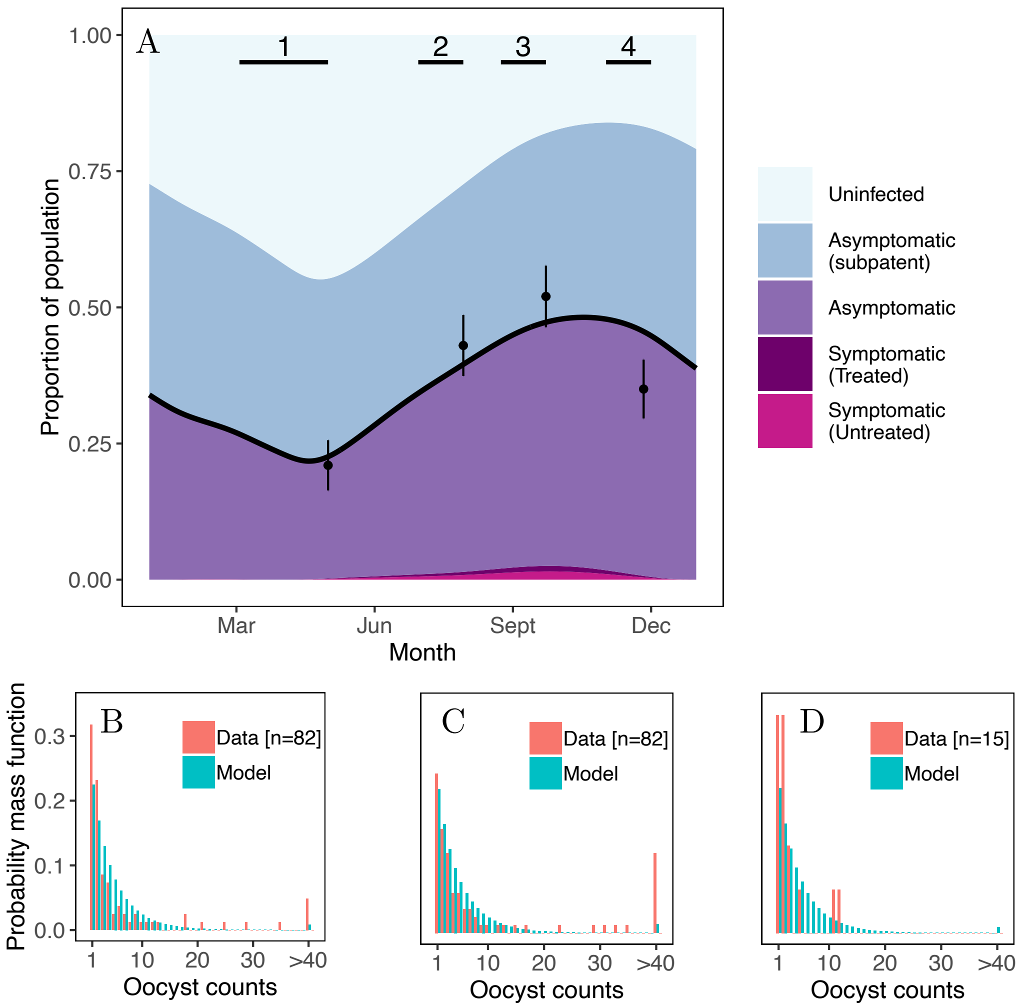

Supplementary Figure 1: Data of malaria prevalence and oocyst counts in naturally infected mosquitoes in Longo (Burkina Faso). Panel A: prevalence data (black dots indicate the proportion, error bars showing the 95% CI) collected at the end of each time period of mosquito collection. These data were used to set the transmission intensity (determined by the value of the entomological inoculation rate, here set to 195 infectious bites per person per year) in the malaria transmission model, utilising data on ITN coverage and levels of insecticide resistance observed in the region. The black curve shows model-estimated prevalence by microscopy over time. The four time periods during which mosquitoes were collected are indicated by the horizontal black lines on this panel. No oocyst-positive mosquitoes were found in the first collection period, due to a combination of a lower malaria prevalence in humans, and a smaller mosquito population present in the study site during the dry season. Panels B, C, and D display the distribution of oocyst counts observed in infected mosquitoes (red bars) in collection periods 2, 3, and 4 and predicted by the best-fit model (turquoise bars). The model was fitted by attributing distinct distributions of oocyst counts for the four infectious states in the model (summarised in Figure 3D and shown in full in Figure S3).

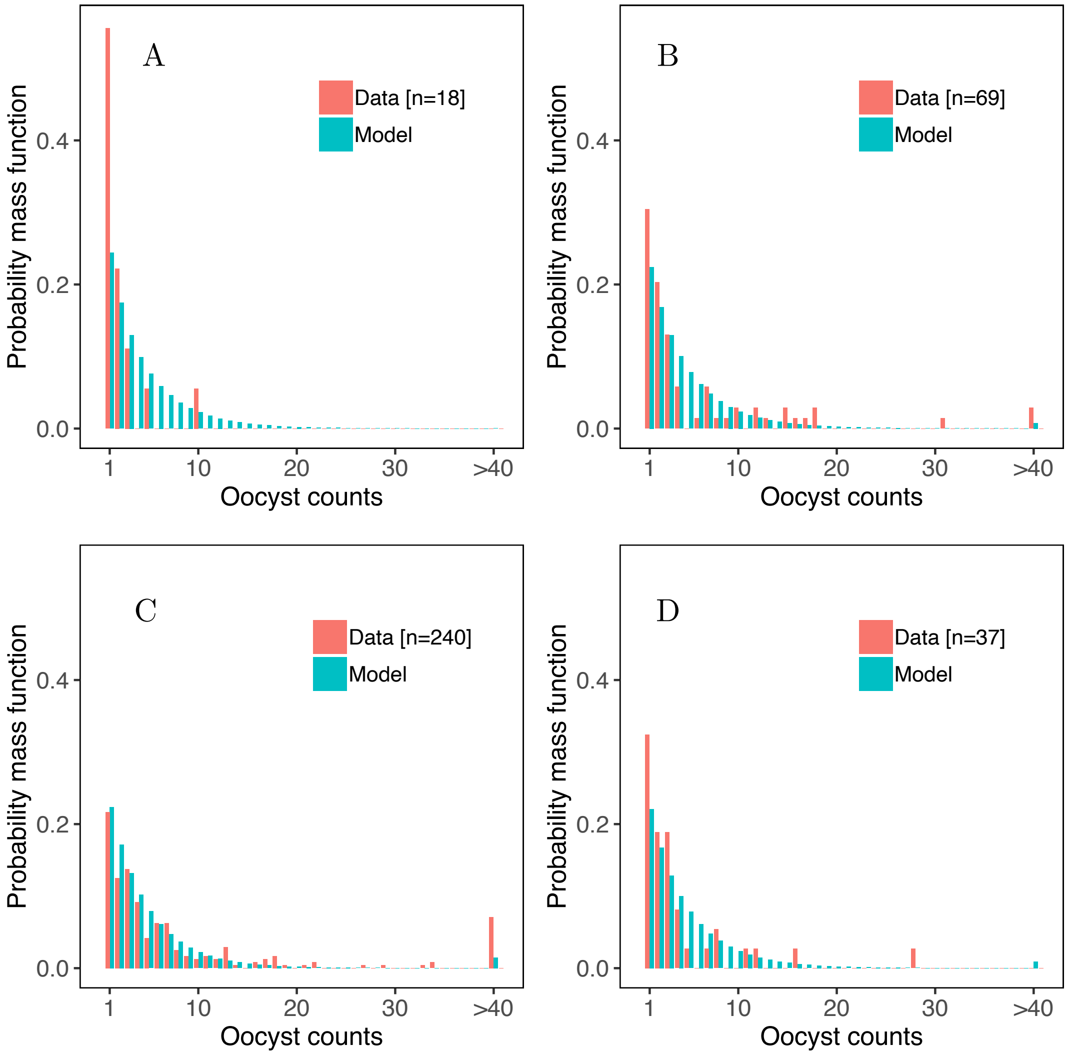

Supplementary Figure 2: Oocyst counts found in naturally infected mosquitoes in Klesso (Burkina Faso). The oocyst data obtained from the four periods (indicated by the horizontal lines at the top of the main panel of Figure 3 in the main text) of mosquito collection in Klesso (time period 1, panel A; time period 2, panel B; time period 3, panel C; time period 4, panel D). The data (red) is compared to the best-fit model (turquoise). Panels C and D are also shown in Figure 3 (panels B and C, respectively) in the main text, but are included again here for the convenience of the reader.

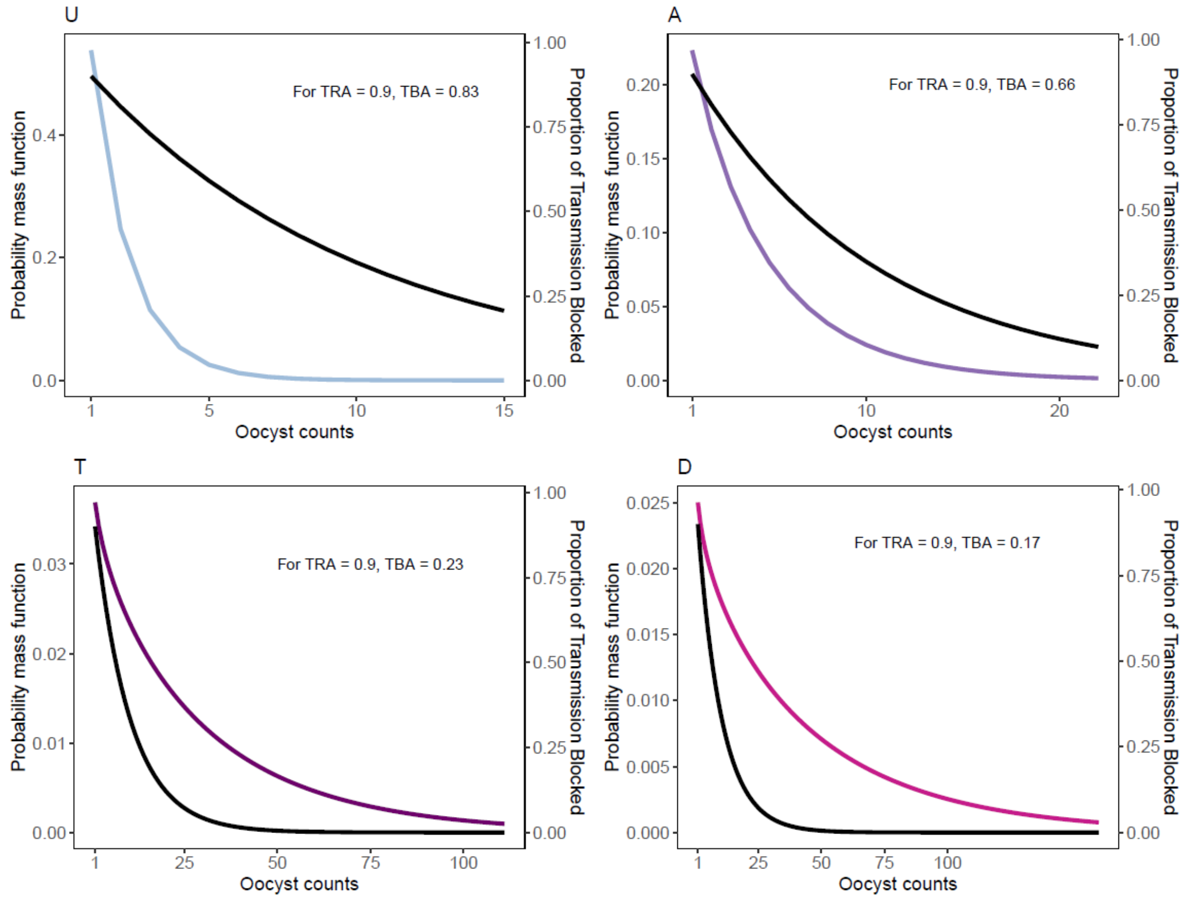

Supplementary Figure 3: Oocyst counts associated with each of the four infectious states. The best-fit distributions (coloured lines) for the oocyst counts associated with each of the four infectious states in the transmission model. The states (as indicated on each panel) are untreated symptomatic disease (D); treated symptomatic disease (T); asymptomatic infection (A); submicroscopic asymptomatic infection (U) (colours as per Figure 3 and Supplementary Fig. 1). These distributions are summarised in their cumulative form in Figure 3D in the main text. The black line (which is the same curve in each panel) gives the probability (scale on the right-hand axis) of a vaccine with a TRA of 90% successively blocking an infection that, in the absence of vaccine-derived antibodies would have produced a given number of oocysts. For this value of the TRA, the TBA for each state is included on the panel.

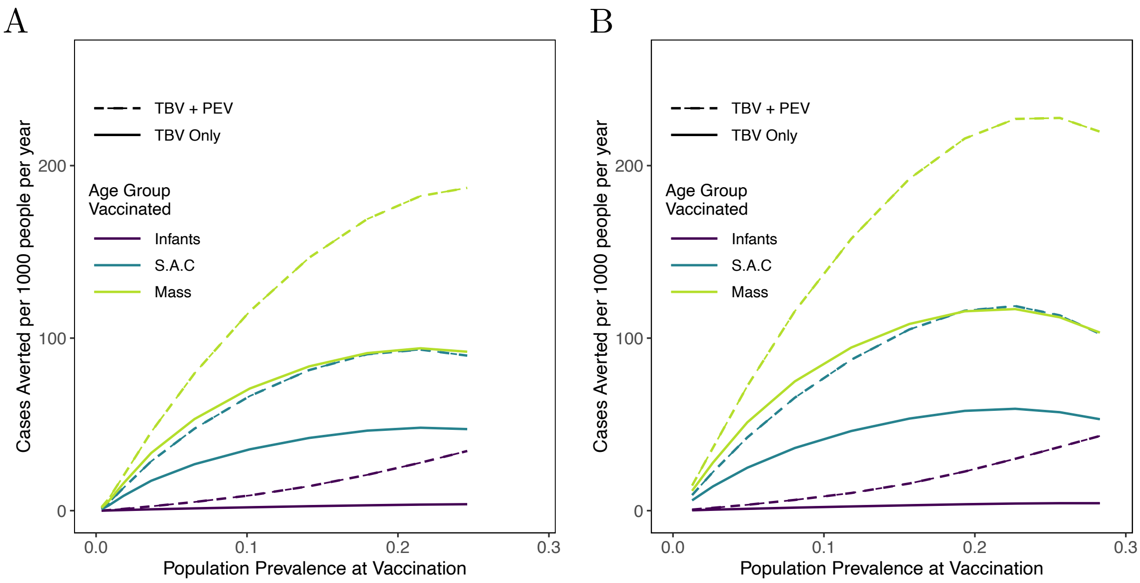

Supplementary Figure 4: Impact of a malaria vaccine at differing transmission intensities. Additional results for the modelling scenarios outlined in Figure 7 in which additional malaria cases are averted by utilising a malaria vaccine (80% coverage of the specified age groups) alongside ITNs. The impact of the vaccine varies depending on the intensity of transmission (which here we represent by the population prevalence prior to the introduction of ITNs). Here we vary the age group targeted for vaccination, and whether a PEV is included. In comparison to a setting in which ITNs are fully effective (panel A), the impact of the vaccination campaign is higher in a setting in which the impact of ITNs is diminished due to high levels of pyrethroid resistance in the mosquito population (panel B).

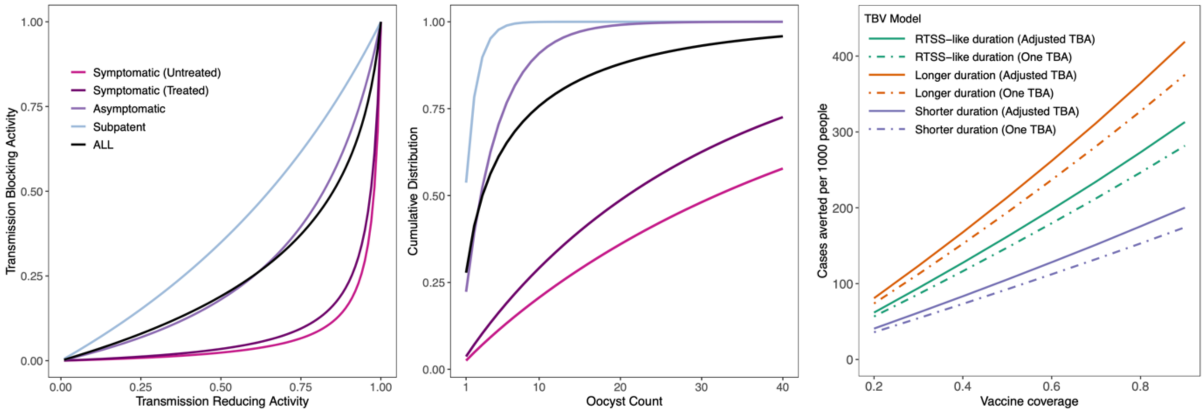

Supplementary Figure 5: Modelling results obtained for a simplified oocyst model. In the results presented in the main text, we assume that mosquitoes become more heavily infected after feeding on humans with symptomatic malaria infections, compared to asymptomatic ones. This means that transmission from symptomatic humans is harder to block. Here we compare that model with a simpler one, in which we relax this assumption, fitting only a single distribution to the oocyst count data. In the left panel, we show the relationship between TRA and TBA obtained for this case (the black line), compared to the four relations obtained in Figure 3 (coloured lines). The middle panel shows the corresponding cumulative distribution of oocyst counts obtained. In the right panel, we repeat the modelling exercise shown in Figure 4, in which we explored the impact of a mass vaccination campaign with a TBV, varying the vaccine coverage and the duration of vaccine activity. We find that the more complicated model (solid lines), with four relations between TRA and TBA for the different presentations of malaria described in the transmission model, predicts a higher number of cases averted than the simpler model (dot-dashed lines). We discuss the reasons for this in the main text.

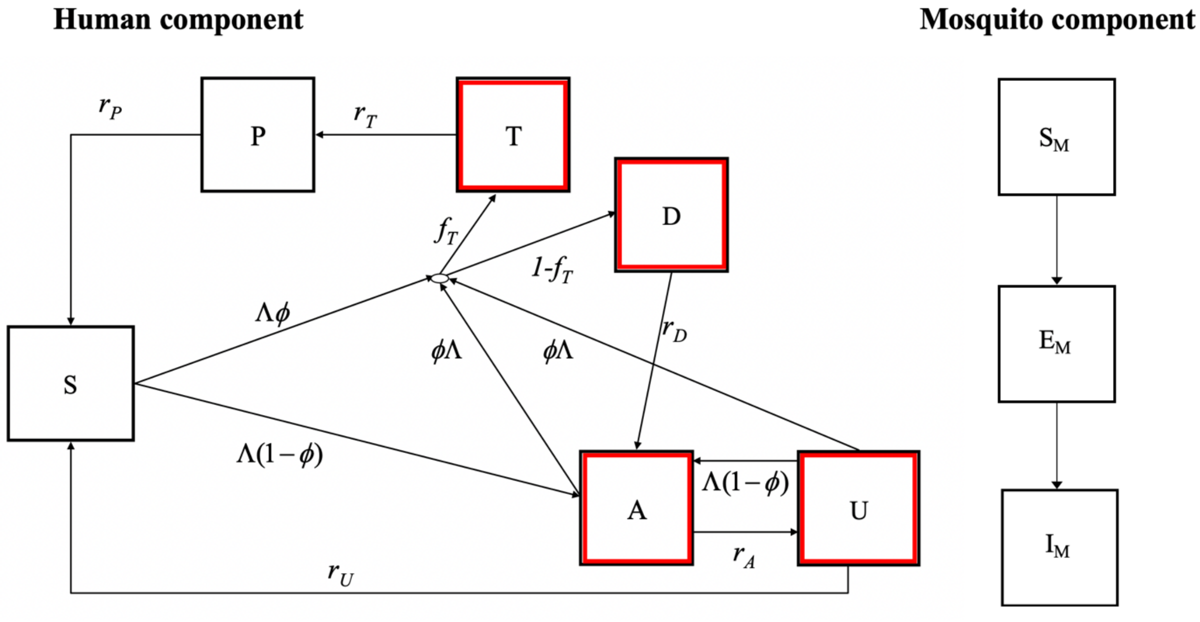

Supplementary Figure 6: Diagram illustrating the structure of the malaria transmission model. The estimated public health impact of a malaria vaccine was generated using an age-structured model of malaria transmission due to Griffin et al. (22,27), which tracks the malaria status of human population, as well as that of the mosquito population. The human population is described using six states: *S* (susceptible), *T* (treated symptomatic infection), *D* (untreated symptomatic infection), *P* (protected, due to drug prophylaxis) *A* (asymptomatic infection), *U* (asymptomatic sub-patent infection). The rate at which humans acquire new infections is determined by Λ, the force of infection, and a proportion (ϕ) of new infections are symptomatic. Parameter f_t indicates the proportion of symptomatic infections that are treated. The rates r_T, r_D, r_A, and r_U, define the mean duration of each infection stage, and r_P governs the duration of prophylaxis due to drug treatment. As indicated by the red outlines, humans carrying an infection can infected feeding mosquitoes, which are described as S_M (susceptible), E_M (exposed, not yet infectious), and I_M (infectious). A transmission-blocking vaccine acts to reduce the probability that a mosquito becomes infected after taking a bloodmeal from an infected human (again signified by the red outline). This figure was adapted by the authors from the one in Ref. (22).

Supplementary Methods

Describing the two malaria vaccines

We commence this section by summarising the RTS,S modelling work due to White et al. (41). Here we will utilise their model of the RTS,S vaccine, and also use it to motivate a model of the duration of activity for a hypothetical TBV. In White et al. (41), the RTS,S vaccine's efficacy against infection, $V(t)$, has the form:

$$V(t) = V_{max}\left[1 - \frac{1}{1+\left(\frac{CS(t)}{\beta}\right)^{\alpha}}\right], \tag{S1}$$

where $CS(t)$ is the antibody titre t days after vaccination, V_{max} is the maximum efficacy of the vaccine, and parameters α and β determine the shape and scale of the dose-response curve. The impact of vaccination with this vaccine is incorporated in the transmission model by reducing the per-bite probability of become infected by an infectious mosquito by a factor of $(1 - V(t))$. After vaccination, antibody titres are assumed to reach their peak value, then wane over time according to a bi-phasic exponential model:

$$CS(t) = CS_{peak}\left(\rho_{peak}e^{-r_s t} + (1-\rho_{peak})e^{-r_l t}\right). \tag{S2}$$

In the trial, some participants received a booster dose 18 months later. Antibody dynamics observed after the booster dose were modelled similarly:

$$CS(t) = CS_{boost}\left(\rho_{boost}e^{-r_s(t-t_{boost})} + (1-\rho_{boost})e^{-r_l(t-t_{boost})}\right). \tag{S3}$$

Throughout this work, we use the parameter values obtained for the older cohort (enrolled at age 5-17 months) of the randomised controlled phase 3 trial (see Table S2). For hypothetical antibody dynamics observed following vaccination with a TBV, $\tau(t)$, we use a very similar model

$$\tau(t) = \tau_{peak}(\rho_{TBV}e^{-r_s t} + (1-\rho_{TBV})e^{-r_l t}). \tag{S4}$$

For simplicity, we assumed identical response to vaccination following a booster dose, as for the primary dose, using $\rho_{TBV} = 0.7$ throughout. For τ_{peak}, we selected a value within the range of values observed in Ref. (10) ($\tau_{peak} = 22$). We retain the value of r_s and r_l obtained for the RTS,S vaccine. In the modelling scenarios where we explore the impact of a shorter- or longer-lasting vaccine activity, we simply halved or doubled these values.

For the TRA of the TBV vaccine considered in this work, we utilised a model fitted by Bompard et al. (10), who estimated the TRA as a function of titre via direct membrane feeding assay using antibodies produced in mice. The model has the following form:

$$TRA = \frac{(\tau/\mu)^{\gamma_1'}}{(\tau/\mu)^{\gamma_1'}+\gamma_2'}, \tag{S5}$$

where τ is the antibody titre, and the parameter values are: $\mu = 12.63$, $\gamma_1' = 2.50$, and $\gamma_2' = 0.06$.

Description of the transmission model

In order to estimate the public health impact of introducing the malaria vaccines into a malaria-endemic population, we utilised an existing model of malaria transmission due to Griffin et al. (22,27). A schematic of the model is displayed in Figure S6, which shows both the human and

mosquito components. This age-structured model, which allows for the development of naturally acquired immunity in populations living in malaria-endemic settings, has been calibrated to data from a range of settings across Africa. Denoting age by a, the human-component of the model can be written mathematically using partial differential equations in the following form:

$$\begin{aligned}
\frac{\partial S}{\partial t}+\frac{\partial S}{\partial a}&=-\Lambda(t-d_E)S(t)+r_P P(t)+r_U U(t)\\
\frac{\partial T}{\partial t}+\frac{\partial T}{\partial a}&=\phi f_T\Lambda(t-d_E)(S(t)+A(t)+U(t))-r_T T(t)\\
\frac{\partial D}{\partial t}+\frac{\partial D}{\partial a}&=\phi(1-f_T)\Lambda(t-d_E)(S(t)+A(t)+U(t))-r_D D(t)\\
\frac{\partial A}{\partial t}+\frac{\partial A}{\partial a}&=(1-\phi)\Lambda(t-d_E)(S(t)+U(t))+r_D D(t)-\phi\Lambda(t-d_E)A(t)-r_A A(t)\\
\frac{\partial U}{\partial t}+\frac{\partial U}{\partial a}&=r_A A(t)-r_U U(t)-\Lambda(t-d_E)U(t)\\
\frac{\partial P}{\partial t}+\frac{\partial P}{\partial a}&=r_T T(t)-r_P P(t)
\end{aligned} \tag{S6}$$

The human population is described using six states: *S* (susceptible), *T* (treated symptomatic infection), *D* (untreated symptomatic infection), *P* (protected, due to drug prophylaxis) *A* (asymptomatic infection), and *U* (asymptomatic sub-patent infection). A full list of parameter definitions is provided in Supplementary Table 3.The rates r_T, r_D, etc. define the mean duration of each infection stage (Figure S6). The force of infection upon the human population at time t is denoted by Λ, with d_E indicating the delay between being bitten by an infected mosquito and developing a blood-stage infection. The probability that, after becoming infected, an individual becomes symptomatic is given by ϕ. Parameter f_t is the probability of receiving drug treatment for a newly acquired symptomatic infection, which for the settings considered here was set to 0.4. We used estimates of treatment coverage in African children under 5 years old as a proxy for treatment coverage of the population. Estimates for treatment coverage in Burkina Faso at the time that the mosquitoes were collected (2014) were estimated from national surveys, collected in Bennett et al. (51). Rather than solving these partial differential equations, the human population is discretised into age groups and the resulting system of ordinary differential equations is solved numerically.

The rate at which people receive mosquito bites is age-dependent, due to body size. In the model this is described by ψ where

$$\psi(a)=\left(1-\rho\exp(-a/a_0)\right), \tag{S7}$$

where $1-\rho$ is the relative biting rate at birth compared to adults, a_0 determines the time-scale of the increase in biting rate with age. In the model, the biting rate is also heterogeneous across the population. It is assumed that each person has a relative biting rate ζ, which persists over their lifetime, having a log-normal distribution between people with a mean of 1 i.e.

$$\log(\zeta)\sim N(-\sigma^2/2,\sigma^2)\,. \tag{S8}$$

This mean that the force of infection at age a and time t, $\Lambda(a,t)$, is given by

$$\varepsilon(a,t) = \varepsilon_0(t)\zeta\psi(a)$$
$$\Lambda(a,t) = \varepsilon(t,a)b, \tag{S9}$$

where ε_0 is the EIR as measured for adults at time t, $\varepsilon(a,t)$ is the EIR for an individual of age a at time t, and b is the probability of human infection if bitten by an infectious mosquito.

To describe the generation and impact of naturally acquired immunity, several auxiliary functions are used. In the model, immunity acts to (i) reduce the probability that receiving an infectious bite results in a blood-stage infection (I_B); (ii) reduce the likelihood of becoming symptomatic after acquiring a new blood-stage infection (maternal and acquired clinical immunity, I_{CM} and I_{CA} respectively); (iii) reduce the likelihood of a blood-stage infection being detected by microscopy (I_D). The exposure-driven aspects of immunity, I_B , I_{CA}, and I_D, all have a similar form,

$$\frac{\partial I_B}{\partial t} + \frac{\partial I_B}{\partial a} = \frac{\varepsilon}{\varepsilon u_B + 1} - \frac{I_B}{d_B}$$
$$\frac{\partial I_{CA}}{\partial t} + \frac{\partial I_{CA}}{\partial a} = \frac{\Lambda}{\Lambda u_C + 1} - \frac{I_B}{d_C}$$
$$\frac{\partial I_D}{\partial t} + \frac{\partial I_D}{\partial a} = \frac{\Lambda}{\Lambda u_D + 1} - \frac{I_D}{d_{ID}} \tag{S10}$$

where in each case the constant u represents the time during which immunity cannot be boosted after receiving a previous boost and the rate d governs the duration of immunity. The probability of an infectious bite leading to a blood-stage infection is given by

$$b = b_0\left(b_1 + \frac{1-b_1}{1+(I_B/I_{B0})^{\kappa_B}}\right) \tag{S11}$$

Where I_{B0} and κ_B are scale and shape parameters. Maternal clinical immunity I_{CM} is present from birth, and is related to the acquired-immunity of a 20-year-old in the population by a constant P_M . This immunity decays at rate $1/d_M$. The probability that a newly acquired blood-stage infection becomes symptomatic is given by ϕ , which has the form:

$$\phi = \phi_o\left(\phi_1 + \frac{1-\phi_1}{1+((I_{CA}+I_{CM})/I_{C0})^{\kappa_C}}\right) \tag{S12}$$

Where I_{C0} and κ_C are scale and shape parameters. As immunity can act to suppress parasite density, it can lead to blood-stage infections becoming sub-patent. In the model the probability that an asymptomatic infection (state A) will be detectable by microscopy is

$$q = d_1 + \frac{(1-d_1)}{(1+((I_D / I_{D0})^{\kappa_D} f_D))}$$

(S13)

Where I_{D0} and κ_D are scale and shape parameters, and f_D is a function of age given by $f_D = 1-(1-f_{D0})/(1+(a/a_D)^{\gamma_D})$ with parameters f_{D0}, a_D, and γ_D. The lower probability of detection also reduces onward infectivity in the model, as it is assumed to be due to lower parasite density. While the infectivity associated with state *D* (c_D), T (c_T), and *U* (c_U) are constant (i.e. independent of levels of naturally acquired immunity), in state *A* infectious is given by $c_U + (c_D - c_U)q^{\gamma_1}$. Parameter γ_1 was estimated during the model fitting process in Ref. (27) (see Supplementary Figure 5 of Ref. (27)).

The mosquito component of the model is much simpler than the human one. Mosquitoes can move between three different states: Susceptible (S_M), exposed (E_M), and infectious (I_M):

$$\frac{dS_M}{dt} = -\Lambda_M S_M^v + \beta(t) - \mu S_M$$
$$\frac{dE_M}{dt} = \Lambda_M S_M - \Lambda_M(t-\tau_M) S_M(t-\tau_M) P_M - \mu E_M \qquad \text{(S14)}$$
$$\frac{dI_M^v}{dt} = \Lambda_M(t-\tau_M) S_M(t-\tau_M) P_M - \mu I_M$$

where $P_M = \exp(-\mu\, \tau_M)$ is the probability that a mosquito survives the period from acquiring infection until sporozoites appear in the salivary glands (τ_M, extrinsic incubation period), μ is the adult death rate, Λ_M is the force of infection acting on mosquitoes and $\beta(t)$ is the time-varying emergence rate. This emergence rate which determines the vector-to-host ratio is adjusted to determine endemicity at the different transmission sites. For Klesso and Longo, the average value of $\beta(t)$ over the course of a year was 27.2 day^{-1} and 10.9 day^{-1} respectively (the size of the mosquito population in the model is expressed relative to that of the human population). The emergence rate is determined by the overall size of the mosquito population. For the full details of the larval-stage of the mosquito model, see Ref. (45). The human adult EIR is given by $\varepsilon = \frac{\alpha\, I_M}{\omega}$ where α is the rate at which a mosquito takes a human blood meal and ω is the normalizing constant for the biting rate over ages, with a population age distribution of $\eta(a)$,

$$\omega = \int_0^\infty \eta(a)\psi(a)\, da \,. \qquad \text{(S15)}$$

The force of infection acting on mosquitoes is the sum of the contribution to mosquito infection from the different human infectious states,

$$\Lambda_M(t) = \frac{\alpha}{\omega} \int_\zeta \int_a \zeta \psi(a) \big(c_D D(\zeta, a, t - t_l) + c_T T(\zeta, a, t - t_l) + c_A A(\zeta, a, t - t_l) + c_U U(\zeta, a, t - t_l) \big) \, da \, d\zeta,$$

(S16)

where c_D, c_T, c_A and c_U are the onward infectivity to mosquitoes of these different states and t_l is the time-lag between parasitaemia with asexual parasite stages and gametocytemia (infectivity to mosquitoes) to account for the lag in gametocyte development characteristic of *P. falciparum*.

<u>The impact of ITNs on the mosquito population</u>

The impact of ITNs upon the mosquito population is to induce mortality and reduce the probability of their successfully acquiring a blood meal. Note that they can only prevent, at maximum, the proportion of mosquito bites that take place whilst people are in bed. Following a recent study of the feeding behaviour of Anopheles mosquitoes across Africa (35), we set this proportion to 0.79. Of the mosquitoes that would have acquired a blood-meal in the absence of the ITN, only a proportion, s_N, do in its presence. The remainder are either repelled (proportion r_N), or killed (proportion d_N), with $s_N + d_N + r_N = 1$. The efficacy of the net decays from its optimal value (indicating below by the subscript 0) over time as follows:

$$r_N(t) = (r_{N0} - r_{NM}) \exp(-\gamma_N t) + r_{NM}$$
$$d_N(t) = d_{N0} \exp(-\gamma_N t)$$
$$s_N(t) = 1 - d_N(t) - r_N(t)$$

(S17)

where γ_N is the half-life (in years) of the insecticide. The inclusion of parameter r_{NM} indicates the ITN still provides some protection (i.e. a physical barrier) against mosquitoes in the absence of any insecticide. The numerical values for these parameters are determined by the level of pyrethroid resistance in the mosquito population. The degree of resistance to the insecticide is determined by a bioassay and previous modelling work (48) has translated the bioassay results into the relevant parameters outlined here. For the two study sites in Burkina Faso considered in this work, we use estimates of the bioassay mosquito mortality in these regions in the year of the study (forthcoming work by Lambert et al.) to obtain the desired parameters. For Klesso (in the region of Haut-Bassins, bioassay mortality 30%) the parameter values are: $d_{N0} = 0.450$, $r_{N0} = 0.359$, and $\gamma_N = 1.821$. For Longo (Centre-Est, bioassay mortality 40%) the parameter values are: $d_{N0} = 0.427$, $r_{N0} = 0.372$, and $\gamma_N = 1.485$. Throughout, parameter $r_{NM} = 0.24$. For both sites, a net coverage of 70% was used (47).

Supplementary Table 1: Fitted values for the oocyst distributions associated with the four infectious states in the transmission model. We used a common dispersion parameter, k, in the four negative binomial distribution. Parameters are as described in the Methods (Equations 3 and 4). We note that parameter m is usually equal to the mean of the negative binomial distribution but here it is not as we are using a zero-truncated distribution. The reported values for the means, given in the Results section, have been adjusted to reflect this truncation. Distributions were fitted to the oocyst counts observed in Klesso and Longo (presented in Figures 1, S1, S2), utilising the malaria status of the populations in the two study sites during the four time periods in which data was collected (Figures 1A, S1A). The posterior distribution was obtained using the Metropolis-Hastings algorithm.

Parameter	Prior Distribution	Posterior Distribution (median and 95% credible intervals)
m_U	$N(\mu = 1, \sigma = 1.5)$	0.8 (0.1-3.6)
m_A	$N(\mu = 4, \sigma = 9)$	3.8 (3.3,4.3)
m_T	$N(\mu = 29, \sigma = 8)$	35.0 (22.7,47.4)
m_D	$N(\mu = 60, \sigma = 25)$	46.7 (30.4,60.0)
k	$N(\mu = 3.8, \sigma = 1.5)$	0.9 (0.6-1.2)

Supplementary Table 2: Summary of model of the RTS,S vaccine model, due to White et al. (50). The antibody decay rates presented here, were the ones observed for the older cohort in the multicentre phase 3 trial (children enrolled between 5-17 months old). For the peak antibody titres, we followed the modelling study by Hogan et al. (34), in which the median value from the 11 study sites was used.

Parameter	Description	Value
CS_{peak}	Peak antibody titre following vaccination	621
CS_{boost}	Peak antibody titre following booster dose	277
ρ_{peak}	Proportion of vaccine response that is short lived following vaccination	0.88
ρ_{boost}	Proportion of vaccine response that is short lived following booster dose	0.7
d_s	Half-life of short-lived component of antibody response (days); $r_s = ln(2)/d_s$	45
d_l	Half-life of long-lived component of antibody response (days); $r_l = ln(2)/d_l$	591
α	Shape parameter for dose-response curve	0.74
β	Scale parameter for dose-response curve	99.2
V_{max}	Maximum vaccine efficacy against infection	0.93

Supplementary Table 3: Parameterisation of the transmission model. Parameter values used in the transmission model, as reported in Griffin et al. (27). We have not varied any of these tabulated parameters in this work. Fuller details may be found in the original publication, including the 95% intervals for the prior and posterior distributions and references for the parameters held fixed during the fitting process. For the parameters determined in the fitting process in Ref. (27), the values displayed here are the median values of the posterior distribution. Parameters are dimensionless, unless stated.

Parameter description	Symbol	Fixed or Estimated	Parameter value
**Age and heterogeneity**			
Age-dependent biting parameter	ρ	Fixed	0.85
Age-dependent biting parameter	a_0	Fixed	8 years
Variance of log of heterogeneity in biting rates	σ^2	Fixed	1.67
**Human infection durations**			
Latent period	d_E	Fixed	12 days
Patent infection	$d_A = 1/r_A$	Fixed	200 days
Clinical disease (treated)	$d_T = 1/r_T$	Fixed	5 days
Clinical disease (untreated)	$d_D = 1/r_D$	Fixed	5 days
Sub-patent infection	$d_U = 1/r_U$	Estimated	110 days
Prophylaxis following treatment	$d_P = 1/r_P$	Fixed	25 days
**Infectiousness to mosquitoes**			
With no immunity, or with untreated disease	c_D	Estimated	0.068
After treatment	c_T	Fixed	$0.32c_D$
In sub-patent infection	c_U	Estimated	0.0062

Relates infectiousness to probability of detection	γ_I	Estimated	1.82
**Immunity reducing probability of detection**			
Probability with maximum immunity	d_1	Estimated	0.161
Inverse of decay rate	d_{ID}	Fixed	10 years
Scale parameter	I_{D0}	Estimated	1.58
Shape parameter	κ_D	Estimated	0.477
Duration in which immunity is not boosted	u_D	Estimated	9.45 days
Scale parameter relating age to immunity	a_D	Estimated	21.9 years
Parameter relating age to immunity	f_{D0}	Estimated	0.0071
Shape parameter relating age to immunity	γ_D	Estimated	4.81
**Immunity reducing probability of infection**			
Probability with no immunity	b_0	Estimated	0.590
Maximum relative reduction	b_1	Fixed	0.5
Inverse of decay rate	d_B	Fixed	10 years
Scale parameter	I_{B0}	Estimated	43.9
Shape parameter	κ_B	Estimated	2.16
Duration in which immunity is not boosted	u_B	Estimated	7.20 days
**Immunity reducing probability of infection**			

Probability with no immunity	ϕ_0	Estimated	0.792
Maximum relative reduction	ϕ_1	Estimated	0.00074
Inverse of decay rate	d_C	Fixed	30 years
Scale parameter	I_{C0}	Estimated	18.0
Shape parameter	κ_C	Estimated	2.37
Duration in which immunity is not boosted	u_C	Estimated	6.06 days
New-born immunity relative to mother's	P_M	Estimated	0.774
Inverse of decay rate of maternal immunity	d_M	Estimated	67.7 days
**Parameters for the mosquito model**			
Average lifetime of adult mosquito	$1/\mu$	Fixed	7.6 days
Extrinsic incubation period	τ_M	Fixed	10 days
